# Implementing a clozapine supply service in Australian community pharmacies: barriers and facilitators

**DOI:** 10.1186/s40545-019-0180-3

**Published:** 2019-08-07

**Authors:** Bethany Wilson, Sara S. McMillan, Amanda J. Wheeler

**Affiliations:** 1 School of Pharmacy and Pharmacology, Griffith University, Gold Coast, Australia; 2 School of Pharmacy and Pharmacology, Quality Use of Medicines Network, Menzies Health Institute Queensland, Griffith University, Gold Coast, Australia; 3 School of Human Services and Social Work, Quality Use of Medicines Network, Menzies Health Institute Queensland, Griffith University, Brisbane, Australia; 4 Faculty of Medical and Health Sciences, University of Auckland, Auckland, New Zealand

**Keywords:** Clozapine, Community pharmacy, Schizophrenia, Australia, Treatment burden

## Abstract

**Background:**

Clozapine is the most effective antipsychotic for treatment-resistant schizophrenia, although serious adverse effects such as agranulocytosis and cardiomyopathy limit its use. In July 2015, Australian regulations changed to allow community-based prescribing and supply of clozapine for maintenance therapy. However, there is currently no information on the rate of clozapine services available in Australian community pharmacies, or the factors that influence a pharmacist’s decision to provide, or not provide, a clozapine service, particularly from the perspective of those pharmacists who do not offer this service. This study investigated Australian community pharmacies providing a clozapine supply service and the barriers to, and facilitators of, implementing this service.

**Methods:**

This mixed method exploratory study was conducted in two stages: (1) a brief online survey of community pharmacists Australia-wide; and (2) semi-structured telephone interviews. The survey was conducted between November 2017–January 2018; results were analysed via descriptive statistics. Survey respondents who did not provide a clozapine service were eligible to participate in a telephone interview exploring barriers and facilitators. Interviews were undertaken between December 2017–January 2018 and data analysed thematically.

**Results:**

A total of 265 pharmacists completed the survey; 51.3% (*n* = 136) provided a clozapine service. Consumer demand was a key facilitator (*n* = 18/247; 66.1%) and the main barrier to implementing a clozapine service was a perceived lack of need (*n* = 70/122; 57.4%). Twelve survey respondents were interviewed; while most participants acknowledged that supplying clozapine in community pharmacies would benefit consumers due to convenience, the lack of training and support led to difficulties in service implementation.

**Conclusions:**

Although regulatory changes aimed to improve access to clozapine, it is unclear if they have been successful, or to what degree. Community pharmacists were positive about supplying clozapine in the community but identified a need for training and support to raise awareness of the service so that eligible clozapine consumers can be transitioned to community-based care. Further research is needed about the perceptions of clozapine consumers to determine whether the regulatory changes have resulted in positive outcomes for their independence and quality of life.

## Background

Schizophrenia is a complex and long term mental illness that typically emerges in late adolescence or early adulthood [1]. It is characterised by psychotic symptoms that are broadly divided into positive and negative effects [1]. Individuals with schizophrenia typically experience positive symptoms, including hallucinations, delusions, disorganised speech and behaviour, and negative symptoms such as a flattened affect or reduction in emotional expressiveness, lack of motivation and social withdrawal [1]. Schizophrenia is ranked in the top 20 causes of disability globally and is estimated to affect 21 million people worldwide [2, 3].

Internationally, second-generation (atypical) antipsychotics, with the exception of clozapine, are the first-line treatment options for schizophrenia [1, 4, 5]. It is estimated that 30% of people with schizophrenia continue to experience persistent symptoms of varying severity despite first-line antipsychotic treatment [6]. In Australia, current guidelines recommend clozapine use in the management of treatment-resistant schizophrenia (TRS) where individuals are non-responsive to, or intolerant of, at least two antipsychotic agents other than clozapine [7]. In most countries where clozapine is available, consumers are required to be registered with a haematological monitoring program due to the associated risk of agranulocytosis, a blood dyscrasia affecting white blood cells [1, 8]. In Australia, mandatory and ongoing haematology monitoring for the duration of clozapine treatment is required prior to any clozapine supply being dispensed; a full blood count is needed every week for the first 18 weeks of treatment and then every four-weeks with continuing use. This monitoring program allows consumers at risk of developing agranulocytosis to be identified before it becomes life-threatening, and may be one of the factors involved in reducing associated mortality rates [9].

The difference in mortality between clozapine and other antipsychotic drugs might be attributable to more intensive monitoring during clozapine treatment, increased effectiveness of clozapine, low safety of other drugs, or all these factors.

Internationally, rates of clozapine use for people with schizophrenia vary greatly, with only 2–3% in some areas of the United States of America (USA) [10] and up to 60% in China [11]. Although clozapine prescribing rates have been steadily increasing in Australia [12, 13], data indicates that clozapine is being prescribed at a lower rate than the estimated prevalence of TRS [13]. From dispensing data obtained from the PBS (Pharmaceuticals Benefit Scheme),^1^ it was estimated that 8.3% of people with schizophrenia from Queensland, Australia, were treated with clozapine in 2013 [12].

The global difference in clozapine prescribing rates reflects variations in clozapine registration (i.e. therapeutic indications) and supply regulations, including prescribing, monitoring and dispensing. Clozapine is one of the most commonly used antipsychotics in China and, unlike other countries, is used in the management of a number of psychiatric disorders including mania, treatment-resistant depression and substance abuse (14). A comparison of the provision of clozapine in China, Denmark, Ireland, Japan, The Netherlands, New Zealand (NZ), Romania, the United Kingdom (UK) and the USA, found that the majority of these countries have national prescribing guidelines allowing for its availability in primary care settings [14]. For example, the Netherlands, UK, USA and NZ allow general practitioners (GP’s) to prescribe clozapine [14]. A shared-care arrangement is used in the UK and NZ for clozapine consumers with stable mental state and blood monitoring after the first 18 weeks of treatment [14]. Care is provided by a GP, under the ongoing supervision of a psychiatrist [15], allowing community pharmacies registered with the relevant haematological monitoring program to dispense clozapine [14].

Prior to the 2015 regulatory changes in Australia, people taking clozapine were required to see a hospital-based psychiatrist every three to six months for their regular clozapine prescription and then have this dispensed at the associated hospital pharmacy every four-weeks [16]. These strict supply arrangements were perceived to significantly impact on a consumer’s ability to access clozapine and contributes to underutilisation and indirectly, adding to the treatment burden that is experienced by those with TRS [16]. Treatment burden is a dynamic and multidimensional concept associated with any tasks related to therapeutic treatment independent of illness, including: interference with daily activities, psychosocial and social impacts, time and travel burden, financial burden, and physical burden such as medication side effects [17, 18]. While recent evidence has highlighted that treatment burden may be less of a concern for a small sample of clozapine consumers in Australia [19], in general, treatment burden is known to influence medication adherence and consequently, consumer wellbeing [17, 18].

In an attempt to improve access to clozapine and reduce treatment burden, in July 2015, the Australian Federal Government amended legislation to allow community-based prescribing and dispensing of clozapine [16, 20]. These changes were part of the Highly Specialised Drugs Program, which also permitted HIV antiretroviral medications and hepatitis B medications to be prescribed and supplied in the community. The changes permitted GP’s to prescribe, and community pharmacies to dispense, clozapine for maintenance treatment [16]. Maintenance treatment is characterised by completion of at least 18 weeks treatment under the supervision of a psychiatrist, the clozapine dose is considered stable, and the psychiatrist approves community-based care [7]. Community pharmacies wanting to dispense clozapine are required to register with a haematological monitoring program based on the brand of clozapine supplied [16]; pharmacists are responsible for checking that valid haematological results are within the safe range prior to clozapine supply.

Community pharmacies are readily accessible to most Australians with currently over 5700 community pharmacies [21] and 28,820 fully registered pharmacists [22]. A recent Government Inquiry identified that community pharmacies are generally well distributed throughout urban, rural and remote regions of Australia [23]. For example, the Pharmacy Guild of Australia, the organisation that represents community pharmacy owners, reported that consumers in country areas live, on average, just over six kilometres away from the nearest pharmacy [24]. Pharmacists play a vital role in ensuring the provision of timely access to necessary medicines for all Australians; community pharmacies are well placed to improve clozapine access to consumers in urban, rural and remote areas.

However, there is limited detail about the factors that influence a community pharmacy’s decision to implement a clozapine supply service. In 2016, Gan and O’Reilly [25] explored the attitudes of Australian community pharmacists who were currently providing a clozapine supply service. The authors identified numerous benefits for consumers and pharmacists alike, such as better rapport and relationships between both parties, the provision of holistic care, and increased consumer access to clozapine [25]. Ultimately, transitioning eligible consumers from hospital settings to less restrictive models of care, such as community-based prescribing and dispensing, is expected to provide normality, flexibility, improved quality of life, greater satisfaction and reduced stigma for clozapine consumers [15].

Gan and O’Reilly noted in their study that there was no information on the number of community pharmacies in Australia which provided clozapine since the regulatory changes were implemented [25]. Furthermore, the perspectives of Australian community pharmacists who do not dispense clozapine have not yet been explored. Therefore, the aims of this research were to: i) identify the number of community pharmacies in Australia providing a clozapine supply service; and ii) explore the barriers and facilitators to implementing a clozapine supply service in Australian community pharmacies.

### Study design overview

Similar to Gan and O’Reilly [25], this mixed-method exploratory study was conducted in two stages. Stage One was an online Australia-wide survey, and in Stage Two semi-structured interviews were conducted with community pharmacists who did not provide a clozapine service. Ethical approval was obtained from a University Human Research Ethics Committee (GU Ref No: 2017/909).

## Methods

### Stage one

A brief online survey was used to identify the number of pharmacies who provided a clozapine supply service and the factors that influenced a pharmacist’s decision whether or not to provide this service. Stage One respondents were community pharmacy owners, managers or lead pharmacists as they were likely to be involved in decision-making about implementing a clozapine supply service. Survey development was informed by the limited literature on clozapine supply within community pharmacies [25], and research exploring the uptake of other professional services in similar contexts [26, 27]. Survey piloting was undertaken by three community pharmacists and two research supervisors (SM and AW). To improve response rates, the survey (outlined in Table 1), was designed to take less than 5 minutes to complete using Survey Monkey©, an accessible, user-friendly survey tool able to be completed on a mobile device.

**Table 1:** Stage One Survey

Demographic questions Pharmacy name and participant role (owner, manager or other) Do you provide other professional services in your pharmacy? (Tick all that apply) None Home medicines review (HMR) Residential medication management review (RMMR) MedsCheck and/or Diabetes MedsCheck Diabetes screening trial (as part of the Sixth Community Pharmacy Agreement) Dose administration aids Aboriginal health services Opioid substitution therapy (OST) Harm reduction (sharps/needle care) Pain management Other (please specify) Do you currently provide a clozapine supply service? Yes or No	
*Questions for providers (Yes)*	*Questions for non-providers (No)*
How long have you been supplying clozapine? < 6 months; 6 months–1 year; 1–2 years; 2 + years	Have you been approached by a consumer requesting clozapine supply? Yes or No
How many consumers do you supply clozapine to? Nil; 1–5; 6–10; 11–15; 16+	Have you ever considered providing a clozapine supply service? Yes or No
Did you undertake training to supply clozapine? No - would you like training, if so what? Yes - please specify How do clozapine consumers get referred to your pharmacy? What influenced your decision to provide a clozapine supply service? (select all that apply) -Remuneration -Access to education/training -Pharmacists knowledge of clozapine and clozapine monitoring systems -Relationship with prescribers -Consumer demand for supply -Pharmacist confidence in ability to supply clozapine -Access to consumer notes -Adequate space & resources -Other (please specify)	What factors influenced your decision not to provide a clozapine service? (select all that apply) -Limited financial return -Lack of time -Lack of access to training -Increased resource requirements -Lack of external support/assistance -Clozapine has difficult supply arrangements -Do not believe clozapine should be supplied in the community -Lack of a need to provide service -Difficulties in communication with prescribers -Poor communication about regulatory changes -Limited knowledge of clozapine monitoring requirements -Other (please specify) Are you interested in discussing this further in a 15–20 min telephone interview? Yes or No

A variety of recruitment methods were used to contact the study population because a national electronic database with email contact details for community pharmacies, or pharmacists, was not available. Recruitment methods included: emailing information about the study (with a link to the survey) to professional colleagues and pharmacy banner groups; placing advertisements with a link to the survey in pharmacy newsletters; obtaining advertising support from a professional pharmacy organisation; cold-calling community pharmacies, and snowballing (i.e. respondents were asked to email their colleagues and/or networks with information about the study, and a link to the survey). Additionally, community pharmacy phone numbers were available on separate registers for six of the seven states and territories in Australia^2^ (Queensland was the exception) and other contact information was found through online databases such as the White Pages™, and The Pharmacy Guild of Australia ‘Find a Pharmacy Service’ [28].

A total of 781 pharmacies were contacted by email (*n* = 738) or phone (*n* = 43) during November 2017 to January 2018. Promotional e-mails that included the survey link were used as they did not interrupt a pharmacist’s workflow and allowed greater flexibility to respond during non-work or off-peak hours. In cases where email contact details were not available, pharmacies were contacted by telephone to request the pharmacy email so that the survey invitation could be sent. Completion of the survey was taken as consent to participate. To encourage Stage One participation, an opportunity to win one of five $50 (AUD) gift vouchers was offered.

The research team contacted the two national haematological monitoring programs (ClopineConnect™ and Clozaril Patient Monitoring System™) and State and Territory Health Departments. No data on the number of community pharmacy clozapine supply services were obtained from any of these sources as they were unable to provide this information.

### Data analysis

Descriptive statistical analysis involved the use of Microsoft Excel©; data were summarised using frequencies and percentages.

### Stage two

Semi-structured telephone interviews were undertaken to obtain a more in-depth understanding of the barriers and facilitators to implementing a clozapine supply service in the community pharmacy setting. The semi-structured interview guide (Table 2) was developed from previous research exploring the uptake of professional services in Australian community pharmacies [26, 27]. The interview was piloted with four community pharmacists to assess the suitability and effectiveness of interview questions, technique and timing, and with both research supervisors (SM, AW) to ensure consistency of data collection.

**Table 2:** Stage Two Interview Guide

1. From your survey response, we understand you do not supply clozapine. What did you think about the regulatory changes that allowed clozapine to be accessed in community pharmacies?	
2. Have you considered supplying clozapine in your pharmacy? If yes, why did you choose to not supply?	
3. What are your thoughts/opinions on providing clozapine in the community?	
4. What do you think are the benefits of supplying clozapine in the community (benefits for both health professionals and consumers)?	
5. What do you think are possible issues/concerns supplying clozapine in the community (for both health professionals and consumers)?	
6. What support do community pharmacies need to implement clozapine supply services?	

### Participants and data collection

Respondents who completed Stage One were invited to participate in a semi-structured interview if their community pharmacy did not provide a clozapine supply service. Interviews were conducted via telephone to allow pharmacists Australia-wide to participate and were timed to take approximately 20 min. All interviews were audio-recorded with consent, transcribed verbatim by the interviewer (BW) and de-identified using a unique code.

All transcripts were quality checked by another research team member (SM) to ensure accuracy. Participants were offered a copy of their individual transcript to check meaning or provide further clarification; a $30 gift voucher was posted to them in appreciation of their time. At the interview conclusion, a written debrief was provided to the research team to discuss key ideas and provided the lead author (BW) with an opportunity to reflect on each interview experience.

### Data analysis

The approach to data analysis was descriptive thematic analysis and based on the phases of thematic analysis developed by Braun and Clarke [29], in which themes are derived from the data itself. Interview transcripts were read and re-read and general themes were identified by the researcher (BW). Categories were then generated, which were confirmed by a research supervisor (SM). The research team as a whole then reviewed themes and categories to ensure reliability of the analysis.

## Results

Verbatim quotes from interviews and survey text are interspersed throughout this section to contextualise the data.

### Stage one

Of the approximately 5700 community pharmacies in Australia [21], this survey was completed by 265 (Table 3), representing 4.6% of community pharmacies in Australia. As a variety of recruitment methods were used, and the research team was unable to track individual uptake, the overall response rate could not be calculated. The majority of respondents were working in independent pharmacies (*n* = 156/262; 59.5%) and were mostly pharmacy owners (*n* = 112/264; 42.4%). Just over half of the responses were from Queensland and New South Wales. This response rate is consistent with the representation of 50% of all Australian community pharmacies in these two states [30, 31]. A total of 136 respondents (51.3%) provided a clozapine supply service in their pharmacy at the time of the survey. Table 3 shows that South Australia had the highest number of respondents providing the service, and Northern Territory had the smallest. Provision of other professional services in the pharmacy was found to be similar, irrespective of whether the pharmacy provided a clozapine supply service or not (Table 3).

**Table 3:** Survey Respondent Characteristics

Variable	n	%	n	%	n	%
*State/Territory* (*n* = 265/5700)			*Clozapine Service* ^a^ (n = 136)		*No Service*^a^ (n = 129)	
Queensland	69	26.0	33	47.8	36	52.2
New South Wales	67	25.3	41	61.2	26	38.8
Victoria	45	17.0	22	48.9	23	51.1
Western Australia	42	15.9	18	42.9	24	57.1
South Australia	22	8.3	15	68.2	7	31.8
Australian Capital Territory	7	2.6	5	71.4	2	28.6
Tasmania	5	1.9	1	20.0	4	80.0
Northern Territory	3	1.1	–	–	3	100.0
Unknown	5	1.9	1	25.0	4	75.0
*Professional Pharmacy Services provided* (*n* = 263/265)			*Clozapine Service* ^b^ (n = 136/136)		*No Service* ^b^ (n = 127/129)	
Dose Administration Aids	254	96.6	132	97.1	122	96.1
MedsCheck^c^	234	89.0	119	87.5	115	90.6
Home Medicines Reviews^d^	169	64.3	94	69.1	75	59.1
Opioid Substitution Therapy	135	51.3	76	55.9	59	46.5
Harm Reduction^e^	133	50.6	71	52.2	62	48.8
Pain Management^f^	86	32.7	44	32.4	42	33.1
Diabetes Screening Trial^g^	58	22.1	29	21.3	29	22.8
RMMR^h^	41	15.6	26	19.1	15	11.8
Aboriginal Health Services^i^	32	12.2	14	10.3	18	14.2
None	3	1.1	2	1.5	1	0.8
Other^j^	33	12.5	15	11.0	18	14.2

^*a*^ *Percentage across each State or Territory;* ^*b*^ *Percentage of clozapine or non-clozapine supply pharmacies that provide the specified Professional Pharmacy Services;* ^*c*^ *a remunerated service in which a pharmacist reviews a consumer’s medication in a consultation in the pharmacy;* ^*d*^ *an accredited pharmacist conducting a medicine review within a consumer’s home;* ^*e*^ *opioid replacement therapy and needle and syringe program;* ^*f*^ *any service related to pain management, including risk assessment for analgesic misuse;* ^*g*^ *a Sixth Community Pharmacy Agreement research project exploring the clinical and cost-effectiveness of diabetes screening interventions within the pharmacy setting;* ^*h*^ *A Residential Medication Management Review is similar to a Home Medicines Review but within an aged care setting;* ^*i*^ *Services provided in collaboration with Aboriginal Health Services;* ^*j*^ *Other includes immunisations, sleep apnoea management, weight management, HbA1c monitoring, stroke risk assessment, cholesterol screening, iron testing, medical certificates & wound management*

The other professional services most commonly provided by pharmacies were dose administration aids (*n* = 254/263; 96.6%) and MedsCheck [32] which is an in-pharmacy medication review service focused on the quality use of medicines (*n* = 234/263; 89.0%). Three respondents indicated that their community pharmacy provided no professional services and one respondent did not answer this question.

#### Pharmacies providing a clozapine supply service (*n* = 136)

Follow-up questions on barriers and facilitators were answered by 127 respondents (93.4%). Most pharmacies supplied clozapine to five or less consumers (*n* = 80/127; 63.0%) and had implemented the clozapine service more than 2 years prior to the survey (*n* = 68/127; 53.5%). When asked how clozapine consumers were referred to the pharmacy, 39.4% (*n* = 50/127) of respondents stated that this was via local mental health services, followed by local hospitals (*n* = 37/127; 29.1%) or other prescribers (*n* = 31/127; 24.4%). Almost two-thirds of respondents (*n* = 73/121; 60.3%) had undertaken clozapine training prior to service implementation, which was primarily hosted by a local hospital or a clozapine manufacturer. Of those respondents with no training prior to supplying clozapine (*n* = 48/121; 39.7%), just less than half (*n* = 23;47.9%) did not believe that training was necessary. Nearly half of this group indicated that pharmacist knowledge and confidence was a facilitator for service provision. Twenty-two of the respondents who had not completed any training reported that they wanted to be trained; six preferred an online training format and four specifically wanted training on how to resolve issues such as missed doses or blood tests. The three remaining respondents were either unsure about the need for training or did not provide a clear response to this question.

The top three facilitators to supply community-based clozapine were i) consumer need/demand for the service; ii) recommendation from a mental health service or hospital; and iii) a pharmacist’s confidence in their ability to safely supply clozapine (Table 4). From the ‘other’ option, three respondents identified the service as a professional benefit as they already packed clozapine into customer Dose Administration Aids and five respondents identified that clozapine supply was part of their job to contribute to care:

“*No reason* [to provide service] *other than a regular customer wanting to access directly through us. I consider it to be a service to the customer as we don't receive any extra funding and it* [dispensing clozapine] *entails a fair bit of paperwork and timing regarding ordering of clozapine brand*” (ID 109; Male, Pharmacy Owner).

**Table 4:** Characteristics of pharmacies that provide a clozapine supply service (n = 127)^a^

Characteristics	n	%		n	%
*Duration of service availability*			*Facilitators for service implementation* ^b^		
< 6 months	9	7.1	Consumer demand	84	66.1
6 months–1 year	11	8.7	Mental health service/hospital suggested	77	60.6
1 year–2 years	39	30.7	Pharmacist confidence to supply clozapine	58	45.7
2 years +	68	53.5	Relationship with prescribers	49	38.6
*Number of consumers*			Pharmacist clozapine & monitoring knowledge	41	32.3
Nil	3	2.4			
1–5	80	63.0	Remuneration	22	17.3
6–10	24	18.9	Adequate space / resources	20	15.7
11–15	6	4.7	Access to education / training	17	13.4
16+	14	11.0	Access to consumer notes	1	0.8
			Other ^c^	17	13.4

^*a*^ *127 respondents answered these questions;* ^*b*^ *Multiple answers were allowed;* ^*c*^ *Other includes regular customers requesting clozapine, the opportunity to contribute to care, previously dispensed clozapine in another setting (internationally or in a hospital setting) and clozapine required for Dose Administration Aids*

#### Pharmacies not providing a clozapine supply service (*n* = 129)

A number of these respondents did not complete all the follow-up questions. Overall three-quarters (*n* = 93/123; 75.6%) had never been approached to supply clozapine and half had not considered providing a supply service (*n* = 62/122; 50.4%). Table 5 presents responses to reported barriers: the most frequently cited was a lack of need (*n* = 64/123; 52.5%) and the most infrequent was that respondents did not believe clozapine should be supplied in a community setting (n = 6). Other issues with implementing a clozapine service included finding information on how to do this effectively, delays from organisation administration in initiating the service and reluctance from local mental health services to refer eligible consumers to their community pharmacy:

**Table 5:** Barriers reported by pharmacies not providing a clozapine supply service (*n* = 122)^a^

*Barriers to service implementation* ^b^	n	%
Lack of need for service (e.g. no clozapine consumers)	70	57.4
Limited knowledge of monitoring requirements	32	26.2
Clozapine has difficult supply arrangements	27	22.1
Limited financial return	25	20.5
Lack of time	23	18.9
Lack of access to training	22	18.0
Lack of external support or assistance	19	15.6
Increased resource requirements	18	14.8
Poor communication about regulatory changes	16	13.1
Difficulties in communication with prescribers	13	10.7
Do not believe clozapine should be supplied in the community	6	4.9
Other ^c^	24	19.7

^*a*^ *122 respondents answered these questions;* ^*b*^ *Multiple responses allowed;* ^*c*^ *Other: included no information on how to implement the service, issues in implementing the service or unaware of the service*

### Stage two

Forty-six respondents (37.4%) identified that they were interested in participating in an interview, however 16 did not provide contact information and 18 did not respond to follow-up contact from the researchers (e-mails or phone calls). In total, twelve respondents (9.3%) were interviewed to explore possible reasons for why they did not provide a clozapine supply service. Of these interviewees, there was equal gender and pharmacy role representation (e.g. owner, manager and dispensing pharmacist); four participants were from New South Wales and Western Australia each, and another four were from Queensland, Northern Territory, Australian Capital Territory, and Victoria. Mean interview length was 14.57 min (range 8.25–24.56 min; median 16.31 min). Participants were coded numerically (1 to 12), i.e. P7 represents the seventh pharmacist interviewed. Five key themes were identified: i) pharmacists’ attitudes, ii) awareness and demand, iii) support, iv) time, and v) safety.

#### Theme 1: attitudes of pharmacists

There was unanimous agreement from interviewees that access to clozapine in a community pharmacy would be beneficial to consumers. The majority stated that transitioning clozapine consumers from hospital to community-based dispensing would increase medication access and adherence by decreasing associated travel and wait times. Two participants referred to an environmental benefit when comparing hospital and community pharmacy settings; specifically the friendlier environment in a community pharmacy. Another participant, who had previously dispensed clozapine in a hospital, spoke about the distress when consumers needed to wait for their medication in the busy hospital setting:

*“It’s* [community pharmacy] *a bit more sort of, personal, they wouldn’t have to be speaking through a bulletproof glass window, you know, through a speaker, which is how it is at the hospital….Outpatient dispensary was already quite busy so they* [clozapine consumers] *would have to wait for long periods of time and I think that didn’t help their agitation in those moments”* [P9; Male, Pharmacist]

Other benefits noted from community pharmacies dispensing clozapine included improved medication adherence, increased time for safety and monitoring checks by pharmacists, improved integration of clozapine consumers into the community, and relieving the burden on consumers of collecting multiple medications from different locations:

*“…if they take clozapine, sometimes they forget to tell the pharmacist that they’re on clozapine, and many medications may interact with that. So it’s good to have it in one spot, one place.”* [P1; Male, Pharmacy Owner]

Despite agreement that community-based dispensing would benefit consumers in various ways, pharmacists attitudes towards their role in supplying clozapine differed. Some acknowledged the importance of the community pharmacy’s involvement in mental health and believed that supplying clozapine would increase community engagement and provide variety in their dispensing role. Some participants’ acknowledged the level of disability associated with schizophrenia and consequently the level of care required for this consumer group:

“…*relationships are extremely important. They need time, care and trust”* [P10; Female, Pharmacy Owner]

*“…you’ve got to give these people a chance…in terms of their mental health, you’re talking the worst of the worst and it’s very hard to treat and they can be very unpredictable so, I guess that’s why they are on the clozapine”* [P9; Male, Pharmacist]

Three participants expressed concerns about the potential for aggression or conflict if they implemented a clozapine supply service in their pharmacy. Two interviewees compared clozapine consumers to those receiving opioid substitution therapy (OST), with respect to safety concerns and the potential for these consumers to become aggressive. One participant, who provided OST, expressed their concerns in the event they were the sole pharmacist on-duty:

*“…the kind of patient we get in for clozapine you have to be careful of them, like you should be aware of the type of patient you’re dealing with”* [P1; Male, Pharmacy Owner]

However, in spite of expressed safety concerns, all three participants stated they would implement a clozapine supply service if there was a demand; ensuring that steps and processes were put in place to minimise and manage conflict:

*“…if their blood tests don’t meet, or it falls outside the window, or when they last requested a script, then they will have to go back and see the prescriber then that is something that is going to happen. There is no just giving a three-day* [clozapine] *supply*^3^ *or let’s forget about it this time. If you have rules in place to ensure that this is what’s going to happen up front then you can avoid those issues”* [P4; Male, Pharmacist]

Overall, there appeared to be a tension between protecting business interests and providing an important health service for clozapine consumers. While some participants were of the opinion that supplying clozapine was their responsibility as health professionals, others were concerned about how the service would affect other pharmacy staff, colleagues and customers.

#### Theme 2: awareness and demand

The majority of participants were able to describe what was involved with a clozapine supply service in general terms, with a particular emphasis on the need for regular blood monitoring.

Awareness varied depending on the participant’s previous experience with clozapine. Five participants who were aware of the monitoring requirements stated that they had dispensed clozapine in the past, either in an Australian hospital setting or overseas, while others stated they had heard about it from colleagues or through continuing professional development (CPD) activities. When asked how they would go about implementing the service, participants had difficultly explaining the steps involved, providing limited detail. One participant indicated that they didn’t remember anything about the legislation changing (2.5 years previously), and subsequently had forgotten about this ‘new’ opportunity. Participants explained that whilst they could obtain information from a variety of sources, such as the Pharmaceutical Benefits Scheme [33], the internet, and prescribers, some were unsure where to seek such advice. Four participants asked the interviewer for information about the steps involved in implementing a clozapine supply service.

All interviewees reported, that as far as they were aware, there had been a lack of demand for the service in their local area:

*“Because we just haven’t had anybody who has come in and said ‘*can you provide it for me*...”* [P9; Male, Pharmacist]

When asked if they had ever been approached by a consumer to supply clozapine, only one participant recalled such a request. Ultimately their pharmacy did not provide the service as they had not found the time to look into service implementation.

*“Well I am not aware of a need. No I am sure there is, I am sure we must have patients out there who are being prescribed clozapine but I am not aware of any, and I have not been approached by any.”* [P5; Male, Pharmacy Owner]

Ultimately, this group of interviewees (i.e. who did not provide a clozapine service), were unaware if any of their current customers were taking clozapine. Some pharmacists assumed that because they hadn’t specifically been asked, it was unlikely that there was any demand for this professional service.

#### Theme 3: support

To support the uptake of a clozapine supply service, all participants requested further training to ensure pharmacy staff (i.e. pharmacists, pharmacy assistants and dispensing technicians), were competent to supply this medication. The majority of participants wanted a multifaceted approach to training with involvement from local hospitals, pathology services, pharmaceutical companies, prescribers and professional organisations. One participant wanted the opportunity to observe and learn from other clozapine dispensing pharmacists:

*“I think it* [training/support] *could be online with maybe a visit from someone who is, you know, experienced in it, and just you know, knows the kind of pitfalls”* [P10; Female, Pharmacy Owner]

Participants also wanted specific training on the different brands of clozapine (Clopine™ and Clozaril™), and the associated haematological monitoring systems, including how to respond if the dedicated monitoring software crashed, to ensure a safe and timely supply of clozapine.

One participant explained that their pharmacy had experienced delays in implementing the service due to issues at their local hospital. This participant was interested in supplying clozapine and had sought advice and support from their local mental health service, however, they had been prevented from initiating the service until the hospital created a clozapine dispensing protocol:

*“…that’s the only thing that’s holding me up at this point and they* [hospital] *won’t let me complete my training or train all my staff until they’ve got it all* [protocols].”^4^ [P3; Female, Pharmacy Manager]

One participant who had previously implemented the service, but later stopped because of apparent lack of demand, raised concerns about pharmacists maintaining competence given that pharmacists dispensed clozapine infrequently. For example, remembering the administrative tasks and how to use the associated monitoring software were specific concerns:

*“…given that we haven’t had any scripts, I couldn’t see that we going to get in a scenario where we were seeing enough that everyone was going to be on top of it regularly. Like if you’ve got one patient, that you’re seeing once a fortnight, it’s not enough that everyone really knows what they’re doing and they are confident with the program. As opposed to if you’ve got half a dozen people where everyone is dispensing clozapine every month, then they are regularly doing it and it’s sort of fresh of mind what the procedure is and how to log on to Clopine Connect*™ *and that sort of thing.”* [P12; Male, Pharmacy Owner]

Participants felt that access to ongoing assistance was important in implementing a clozapine supply service and gaps in knowledge should be addressed to support the uptake in community pharmacies.

#### Theme 4: safety

A few participants were concerned about the adverse effect profile of clozapine, acknowledging that some of these adverse effects can be life threatening. Particular concerns were raised for pharmacists who had not undertaken training (e.g. locums), who might dispense clozapine without checking that blood results were within safe ranges:

*“…if you haven’t fully read up on the information and, and know what to look for to identify like with the neutropenia and things like that, and the warning signs, it is, I guess a bit higher risk to the patient and to the pharmacist…”* [P2; Male, Pharmacist]

One participant stated that although they initially had concerns about the adverse effects of clozapine, after speaking with a local mental health service they were reassured that this medication could be safely supplied by community pharmacists:

*“…you know there’s the side effects and everything is always quite concerning but they* [mental health service personnel] *really, you know assured me that, with all the clients that they’ve had on it, there’s only been one that’s ever had to be removed and it’s been so effective for their patients in really controlling their symptoms…”* [P3, Female, Pharmacy Manager]

Two participants were of the opinion that supplying clozapine in a community pharmacy setting might actually increase consumer safety because of access to more complete medication records. Additionally, four participants believed that it may be safer to dispense clozapine in a community pharmacy as these pharmacists often have more time to spend with their consumers, checking for adverse effects and monitoring any changes:

*“I feel at hospital, doctors and nurses they don’t have time. Then, but also I think pharmacists they are, they are underused professionals. I think we, we have knowledge and we should use our knowledge. And we have time, and more time than other health professionals”* [P6; Female, Pharmacist]

#### Theme 5: time

Time limitations were acknowledged by most participants. Demands on pharmacists time were amplified because of the increased administrative burden associated with supplying clozapine, such as checking blood results immediately prior to dispensing to ensure that the medication was safe for the individual consumer:

*“…just making sure that, for instance, all the test results and all that. There’s a bit of liaising with the doctor and pathology and that sort of thing”* [P8; Female, Pharmacy Manager]

Three participants believed that providing a clozapine supply service would be more time consuming than their regular dispensing role, and consequently felt that they should be financially compensated:

*“…some form of remuneration as well because it does cost money to sort of put these things into place and to pay for the extra resources, staffing resources.”* [P8; Female, Pharmacy Manager]

There appeared to be a notable tension between time as a benefit to consumers versus time as a barrier to pharmacists; while some participants stated that community pharmacists had more time, others were concerned that they had insufficient time to provide a reliable clozapine service:

*“…researching and finding out information, and time, you know, because we’re all so busy and you just don’t get a chance to put these sort of things in place”* [P8; Female, Pharmacy Manager]

*“It was just a huge ordeal just to, to set up a small little program”* [P2; Male, Pharmacist]

One participant recognised the time demands involved with dispensing clozapine because it is often prescribed in quantities which are smaller than those provided in the standard packaged product from the manufacturer. Consequently, pharmacists have to open the original packaging and remove the required quantity of tablets as per prescription instructions.

Overall, some participants acknowledged that the requirements for safely dispensing clozapine were frustrating due to the increased administrative burden, while others felt that clozapine could be more safely supplied in the community setting due to the increased time available from community pharmacists to spend with their consumers.

## Discussion

Recent Australian regulatory changes were intended to improve access to clozapine in community pharmacy settings, and increase consumer independence and quality of life [34]. Ideally, the uptake of clozapine supply services in the community would cover most geographic areas in Australia, and hence, improve equity of access. This study was unable to establish the actual prevalence of clozapine supply within Australian community pharmacies, however preliminary results indicate that there was considerable variability in clozapine supply between States and Territories. None of the three Northern Territory survey respondents supplied clozapine; indicating that, at the time of the study, clozapine was restricted to hospital settings. With just under half of the Northern Territory population living in rural/remote areas, access to a hospital facility dispensing clozapine is practically impossible [35]. As clozapine must be dispensed within specific blood monitoring time-frames (i.e. within 48 h of the most recent blood test to monitor for haematological effects [36]), this restricts the ability of hospitals to easily transport supplies of clozapine to rural/remote facilities for consumer collection within the designated time-frame. This time restriction adds another dimension to a medication already associated with considerable treatment burden for consumers. In order to improve community access to clozapine, dispensing pharmacies need to cover all geographical areas, thereby ensuring equity of access in Australia.

In our study the majority of community pharmacists supported community access to clozapine as a particular benefit to consumers. These results confirm those found in a study by Knowles et al. [37]; where hospital and community pharmacists had positive attitudes toward community-based dispensing of clozapine believing it would help to alleviate burden and improve flexibility and convenience for consumers. However, the participants in our study did not believe that implementing a clozapine service was particularly beneficial, aside from enhanced professional satisfaction for pharmacists and potentially increasing pharmacy customer numbers.

In relation to implementing other professional services in community pharmacies, both in Australia and internationally, lack of time, for example due to increased work demands, has been cited as the most important barrier [38–46]. Interestingly in our study, some participants identified time as a benefit with community pharmacists potentially having more time to spend with clozapine consumers. Although some participants noted time limitations, the majority of concerns were focused on a lack of information/resources about the service rather than delivering the service itself. Notably, access to training and support were found to be the greatest facilitator for clozapine supply by Gan and O’Reilly, who emphasised that further training should be made available to Australian pharmacists [25].

Ultimately, the main barrier to implementing a clozapine supply service in our study was perceived lack of need. Most participants were unaware if any of their current customers were taking clozapine, with some assuming that because they hadn’t been asked, then there was unlikely to be a demand for the service. Consequently, there may have been clozapine consumers living within their communities who they were unaware of. Given the potential for serious adverse effects and drug interactions associated with clozapine, it is essential that all health professionals, including pharmacists, have access to medication records for consumers and undertake medicines reconciliation. An Australian study by Murphy et al. [47] investigated the completeness and accuracy of medication records held by health professionals for clozapine consumers managed in a shared-care programme. Overall, 91.4% (*n* = 32) of clozapine consumers had at least one discrepancy in their medication records,^5^ with the majority (*n* = 127; 73.8%) of discrepancies due to medication omissions [47]. Community pharmacy records had the highest rates of discrepancies, primarily omissions of clozapine, because this had been dispensed in the hospital setting [47]. Therefore, community pharmacies were unable to check and identify any issues with concomitant medications because clozapine was not recorded in their dispensing system [47]. Supplying clozapine alongside all other prescribed medications for a consumer at their nominated community pharmacy would ensure that pharmacists have a complete medication record, thereby improving continuity of care and ensuring the safe and appropriate supply of all medications, including clozapine.

### Policy, practice and research implications

Most respondents currently providing a clozapine service indicated that either a mental health service or hospital had encouraged initiation; almost all consumers were referred to them by an external provider. However, there are currently no Australian national or regional registers/lists of community pharmacies providing a clozapine supply service that consumers or health professionals can refer to. As a result, consumers may be transitioned to hospital-preferred community pharmacies rather than a pharmacy of their own choice, or they may be expected to continue to obtain their clozapine treatment every four-weeks fourfrom the hospital even when they are living in the community. This situation is at odds with the purpose of the 2015 regulatory change to improve equity of access and reduce treatment burden. While noting pharmacists’ concerns about safety, a recommendation from our study is to create an accessible registry of pharmacies supplying clozapine, which could also encompass other highly specialised medication, such as HIV and hepatitis B.

Although the majority of participants identified some of the operational steps and issues involved in a clozapine supply service, there was uncertainty overall as to how to implement the service. Furthermore, some pharmacists were unaware of the 2015 regulation changes, indicating that both healthcare professionals and consumers were not adequately informed of these changes and the associated service requirements. This lack of awareness is not altogether surprising, given that currently, there is no national standardised clozapine training or accreditation process available for Australian pharmacists.

Currently in Australia the community pharmacy as a business must be accredited to supply clozapine, not the individual community pharmacist [34]. Participants in our study raised concerns about untrained pharmacists employed at the pharmacy providing a clozapine service, despite the registration and monitoring requirements that have been put in place to minimise risks to consumers and ensure that clozapine is used as safely as possible. One possible option may be to coordinate joint primary care-based prescriber, pharmacist and secondary care mental health staff training sessions to encourage inter-professional learning and networking, and to ensure that eligible consumers are transferred to community-based care in a fully informed manner. Online training modules should be developed as face-to-face training sessions are often difficult (and expensive) for health professionals working in rural/remote areas. Clearly defined protocols related to dispensing procedures, legal requirements and clinical information also need to be created to support the implementation process for community pharmacists, in line with previous recommendations [25]. Globally, Japan is the only country we could identify that had a standardised training program [14]. Prescribers and dispensers must undergo a two to three hour e-learning program prior to prescribing or dispensing; however, the exact content of the training was not reported [14]. Similar information could be sought from relevant overseas programs such as the UK [48], NZ [49], and from the in-house programs that are delivered by some hospital-based services around Australia (such as in South Australia [50]), to inform the development of standardised Australian clozapine supply protocols. Additionally, training for pharmacy support staff should be developed to ensure that they are aware of the monitoring and registration requirements for consumers prescribed clozapine; we were unable to identify any such training for pharmacy support staff.

During our telephone interviews, some participants compared clozapine supply services to OST, one of the first professional pharmacy services funded by the Australian Government [26]. OST is similar to the clozapine supply service in terms of its overall aim, i.e. to improve treatment access for vulnerable populations [26]. Similar to clozapine supply, OST is a non-compulsory service for Australian community pharmacies; it is estimated that only 40% of community pharmacies currently provide this service, resulting in treatment access issues [51, 52]. Safety and security concerns have been commonly cited as barriers for the provision of OST [26, 44, 53–56], as have pharmacists’ attitudes [57–59]. Similar concerns were raised by our study participants about a clozapine service. This finding is not surprising as mental health consumers typically experience stigma from health professionals, including community pharmacy staff [60, 61]. Literature suggests that education and training significantly improves knowledge and reduces stigma in health professionals [62, 63]. For this reason pharmacy staff may benefit from undertaking Mental Health First Aid training in order to improve their mental health literacy [64].

Larger scale studies are needed to determine the actual uptake of clozapine supply services in Australian community pharmacies since the 2015 regulation changes. Given the inherent difficulty in accessing electronic contact details (e.g. email) to invite community pharmacists to participate in research, further support may be needed from clozapine manufacturers and professional organisations to facilitate such research. For example, the inclusion of clozapine services to the list of other professional services like OST and other mental health support listed in The Pharmacy Guild of Australia ‘Find a Pharmacy Service’ [28]. In addition, this study did not explore the views and experiences of consumers who obtain their clozapine treatment from a community pharmacy, and so future research should focus on this aspect to ensure that the new regulations have had the intended effect of reducing treatment burden, and improving quality of life.

### Study limitations

This exploratory study has a number of limitations. Firstly, the low number of responses (265 representing approximately 5% of community pharmacies in Australia) meant that analysis was limited to descriptive statistics. However, just over half of the responses were from Queensland and New South Wales which is consistent with the representation of 50% of all Australian community pharmacies in these two states [30, 31]. Secondly, it is possible that only pharmacists who were motivated and engaged responded to the survey, although we had an approximately 50:50 split with respondents who currently did, and those who did not, provide a clozapine service. This suggests that the survey was not only completed by pharmacists engaged in providing this new service and possibly more motivated to work with mental health consumers. However, more community pharmacies that did not provide the service may have disregarded the invitations to participate in the belief that the study was not relevant to them. The timing of data collection, throughout the December/January summer holiday period, was also likely to have negatively impacted on the response rate, however we had limited flexibility in relation to the timing of the study as it was restricted by time-frame for the lead researcher completing the research in part fulfilment of her Master of Pharmacy degree. Importantly, the lack of a single electronic database of Australian community pharmacies, with email/phone contact details, meant that we employed a range of recruitment methods and hence it was not possible to calculate an overall response rate. Lastly, participant responses to both survey and interview questions may have been influenced by a tendency to give socially desirable responses, introducing a potential bias.

## Conclusion

To our knowledge, this is the first study to explore the number and perspectives of non-providers of clozapine in Australian community pharmacies. Although regulatory changes aimed to improve access to clozapine, it is unclear if this has actually occurred; further work is needed to identify the actual rate of change. This is particularly important for more rural and remote areas of Australia. While a lack of time and remuneration are well-known barriers to implementing professional pharmacy services, this was not the case for a clozapine supply service; the predominant barrier was a lack of perceived consumer need. Raising awareness of this service to both consumers and other healthcare professionals, would help to ensure that eligible clozapine consumers can be transitioned to trained community-based pharmacy service providers with positive benefits to all concerned.

## Abbreviations

ACT: Australian capital territory
CPD: Continuing professional development
GP: General practitioner
HIV: Human immunodeficiency virus
NSW: New South Wales
NT: Northern Territory
NZ: New Zealand
OST: Opioid substitution therapy
PBS: Pharmaceutical benefits scheme
QLD: Queensland
SA: South Australia
TRS: Treatment –resistant schizophrenia
UK: United Kingdom
USA: United States of America
VIC: Victoria
WA: Western Australia

## Footnotes

1 The Pharmaceutical Benefits Scheme (PBS) is an Australian Government Funded program which subsidises the price of listed medications and medical devices for Australian citizens.

2 Australia consists of several regions: Six states - New South Wales (NSW), Queensland (QLD), South Australia (SA), Tasmania (TAS), Victoria (VIC), Western Australia (WA); and two territories (Northern Territory and the Australian Capital Territory (ACT).

3 The participant is referring to three days’ supply of certain prescription medicines when the consumer does not have a valid prescription (i.e. in cases of urgent need). Clozapine is a Highly Specialised Drug and therefore can not be supplied in this way.

4 While at the time of the study the hospital did not have any protocols for community-based clozapine supply, there were procedures for clozapine supply within the hospital setting.

5 Inconsistencies between two or more medication lists
